# Shaping the synaptic signal: molecular mobility inside and outside the cleft

**DOI:** 10.1016/j.tins.2011.03.002

**Published:** 2011-07

**Authors:** Dmitri A. Rusakov, Leonid P. Savtchenko, Kaiyu Zheng, Jeremy M. Henley

**Affiliations:** 1 Institute of Neurology, University College London, Queen Square, London WC1 3BG, UK; 2 Medical Research Council Centre for Synaptic Plasticity, School of Biochemistry, Medical Sciences Building, University of Bristol, University Walk, Bristol BS8 1TD, UK

## Abstract

Rapid communication in the brain relies on the release and diffusion of small transmitter molecules across the synaptic cleft. How these diffuse signals are transformed into cellular responses is determined by the scatter of target postsynaptic receptors, which in turn depends on receptor movement in cell membranes. Thus, by shaping information transfer in neural circuits, mechanisms that regulate molecular mobility affect nearly every aspect of brain function and dysfunction. Here we review two facets of molecular mobility that have traditionally been considered separately, namely extracellular and intra-membrane diffusion. By focusing on the interplay between these processes we illustrate the remarkable versatility of signal formation in synapses and highlight areas of emerging understanding in the molecular physiology and biophysics of synaptic transmission.

## Introduction

The bulk of information processing in the brain relies on rapid diffusion of small signalling molecules in the extracellular space, principally across the synaptic cleft. Over the past decade it has emerged that lateral movement of receptor proteins in cell membranes is equally important to the functioning of neuronal circuits [1–7]. Indeed, the spatiotemporal profile of released neurotransmitters and the precise membrane localisation of receptors are the two crucial factors that shape synaptic signals, the building blocks of data handling and storage in the brain. Here we review the current state of knowledge and recent experimental advances regarding physiological significance, biophysical constraints and available measures of molecular mobility in the synaptic environment. Focusing primarily on excitatory synapses, we highlight important aspects of their function and use-dependent plasticity which are crucially defined by molecular diffusion, and therefore by the mechanisms that regulate this diffusion.

## Extracellular diffusion inside and outside the synaptic cleft

### Neurotransmitter mobility and a spatiotemporal profile of receptor actions

Once released into the synaptic cleft, or ectopically, neurotransmitter molecules diffuse rapidly away from the release site until they are either biochemically degraded or taken up by surrounding cells. At excitatory synapses, 2000–3000 glutamate molecules are released from a synaptic vesicle [8] whereas only ∼50–100 ionotropic glutamate receptors (that mediate the bulk of transmission) are expressed by individual postsynaptic densities (PSDs) [9,10]. Given that only one or two glutamate molecules are required to activate glutamate receptors, this implies that >90% of released glutamate will escape the cleft even when all its synaptic receptors are fully bound. Rapid escape of neurotransmitter implies that its concentration under the release site drops orders of magnitude over one millisecond, whereas 100–200 nm away from the release site it never rises above very low values [11–13]. Although at first glance this arrangement appears an uneconomic waste of signalling molecules, closer analysis reveals clear underlying adaptive roles.

First, by diffusing away from activated receptors, neurotransmitter molecules ensure rapid decay of synaptic signals and minimum levels of receptor desensitisation. Combined with rapid deactivation of synaptic receptors, such as AMPA-type glutamate receptors, this allows particular synapses to operate at an exceptionally high frequency [14,15]. Another important consequence of rapid signal cessation is to increase the precision with which the cell can detect temporal coincidence of brief signalling events. Indeed, coincidence of neurotransmitter release and dendritic action potentials is a crucial parameter for triggering synaptic changes associated with Hebbian-type learning [16,17] (Glossary).

**Figure 1:**
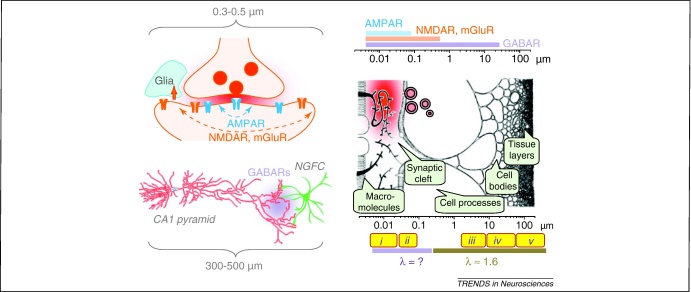
The actions of neurotransmitters in space and time are determined by their extracellular diffusion, uptake and receptor affinity. Left, top: glutamate released into the synaptic cleft activates hotspots of lower-affinity AMPARs but reaches larger pools of higher-affinity mGluRs and NMDARs. Astroglial protrusions enriched in high-affinity glutamate transporters (EAAT1 and EAAT2) could prevent glutamate from activating receptors present where such protrusions are in the way of diffusing glutamate (block arrow, glutamate uptake). Dotted arrows illustrate a comparative range of glutamate actions on different receptors. Left, bottom: inhibitory transmission between a neurogliaform cell (NGFC) and a CA1 pyramidal cell relies on a relatively long-lasting extracellular cloud of GABA (light purple) which activates GABA_A_ and GABA_B_ receptors (GABARs) on the postsynaptic cell [24–26]. Right: extracellular diffusion and receptor actions of neurotransmitters on different scales in the synaptic neuropil (diagram, modified and reproduced with permission from [125]; the width of callouts indicates an approximate dimension range of the corresponding components). Top: approximate distance from the release site at which glutamate can activate various receptors (as indicated; a log scale is used). Bottom: methods that enable extracellular diffusion measurements at difference scales, as indicated by yellow boxes: **(i)** TR-FAIM [126], **(ii)** indirect assessment using viscosity manipulation combined with electrophysiology [46], **(iii)** microfibre-optics measurements [63], **(iv)** point-source fluorescence imaging [18,54,56,62] and photobleaching [58,59], **(v)** iontophoresis of TMA^+^[27,51]. The value of diffusion retardation (λ) in the microscopic range has been determined to be ∼1.6 [18,52–54], but remains unknown at the nanoscopic scale.

Second, rapid escape from the cleft implies that neurotransmitters activate distinct spatial pools of low- and high-affinity receptors. This is best characterised for the excitatory neurotransmitter glutamate which can activate both ionotropic (NMDA, AMPA and kainate subtypes) and metabotropic (mGlu1–8 subtypes) classes of receptors. For example, low-affinity AMPARs are activated by high transmitter concentrations, and these tend to occur within ∼100 nm of the release site [12,13,18] (Figure 1). In this way the bulk of rapid information transfer in the brain occurs via point-to-point (‘wiring’) transmission, with little interference from outside or between individual connections. By contrast, relatively high-affinity receptors, such as NMDA or mGlu receptors (NMDARs or mGluRs), can be activated by an equivalent glutamate release event over a much greater area [19] (Figure 1). In some conditions of intense synaptic activity this diffuse signal can spread [20–22], invoking a concept of ‘volume transmission’ [23]. Recently, the adaptive significance of this mode of transmission has been emphasised by the finding that individual hippocampal neurogliaform cells (NGFCs) signal to CA1 pyramidal cells by releasing the inhibitory neurotransmitter γ-aminobutyric acid (GABA) from axon terminals that make few or no direct synaptic connections with the receiving ‘postsynaptic’ cell [24,25]. Strikingly, this signalling appears to be efficient even when one NGFC fires only a single action potential [26] (Figure 1). This discovery suggests that GABAergic volume transmission could play a more important role in the brain than was previously thought.

### Factors affecting the extracellular mobility of small transmitter molecules

The speed with which small molecules diffuse outside cells within the brain depends on extracellular space connectivity and cellular uptake (or clearance through binding or chemical degradation). The impact of tissue geometry upon extracellular diffusion can be summarised by the extracellular space volume fraction α and tortuosity λ, the latter reflecting an apparent path increase compared to diffusion in a free medium (thus corresponding to a λ^2^-fold reduction in the diffusion coefficient) [27]. Subsequently, λ was suggested to incorporate average viscosity [28] and dead-space extracellular domains that transiently trap diffusing molecules [29]. Several pathological conditions have been associated with changes in α and λ. During severe anoxia or ischaemia the value of α in the cortex drops from 0.20 to 0.05, while λ rises from 1.5 to ∼2.1 [30]. A similar decrease in α has also been reported during cortical spreading depression [4] and, to a lesser extent, during epileptic seizures [31,32] (the latter should not be confused with bulk diffusion changes due to progressive cell loss associated with status epilepticus *in vivo* [33]). However, establishing a causal connection between such changes and the underlying cellular mechanisms remains an important task.

The principal role of uptake is to keep extracellular concentrations of neurotransmitters low, thereby ensuring high sensitivity of synaptic receptors to rapid release events. The time-averaged ambient level of glutamate is estimated to be ∼25 nM in quiescent tissue [34], and this should be below the 0.1% activation level of any known glutamate receptors. Indeed, high-affinity glutamate transporters, which are abundantly expressed in astroglia [35], curtail any long-range actions of glutamate escape even during intense activity [18,36,37]. Much less is known about the transporter efficiency and the prevalence of ambient GABA. In contrast to glutamate, tonic and phasic activation of both synaptic and non-synaptic ionotropic (GABA_A_) and metabotropic (GABA_B_) receptors can be readily detected in different brain regions [38–40]. Together with a prominent example of volume GABA transmission enacted by neurogliaform cells (Figure 1), this suggests that the adaptive roles of local neural circuitries could require distinct GABA receptor subtypes and varied local expression of GABA transporters.

The picture is even more complicated on a sub-micron scale, principally because individual features of the synaptic environment cannot be summated as volume-averaged quantities. For example, although glial transporters rapidly buffer glutamate outside the cleft [36], astroglia approach only 30–40% of an average synaptic circumference [41,42] and tend to be localised on the postsynaptic side [43] of hippocampal synapses. In cerebellar Purkinje cells, synaptic activation of mGluRs is selectively blocked in areas that show high densities of the glutamate transporter EAAT4 [44]. These observations indicate that the non-homogeneous distribution of transporters can leave escape routes for glutamate, thus extending the range of its excitatory actions, but only in certain spatial directions [18] (Figure 1). This suggests that the local synaptic environment can diversify the function of anatomically similar synapses operating in the same anatomical circuitry.

Another factor, first noted decades ago [45], that can affect the mobility of charged neurotransmitters is the local electric field arising around hotspots of ion currents. The electric field (*E*) is classically determined by the current (*I)* flowing across an interface surface (*S)* and the electrolyte resistivity (*R*_*e*_) according to the equation *E* = *R*_*e*_*I/S*. With a typical synaptic current of ∼10–100 pA, a characteristic *R*_*e*_ inside the cleft of ∼150 Ohm·cm (two- to threefold of that in a free medium [18,46] at 37^o^C, see below), and synaptic cleft dimensions of 200–300 nm by 15–20 nm for a single PSD [43,47], the electric field should be in the region of 10^4^ V/m [48]. This strong field is expected to affect the diffusion of negatively charged glutamate molecules in the cleft. Consistent with this, a recent electrophysiological investigation found that the direction and magnitude of postsynaptic currents can alter the intra-cleft dwell-time of glutamate (but not of electrically neutral GABA), thus modifying the excitatory signal waveform at hippocampal synapses [49]. It is an intriguing and open question whether such phenomena play a use-dependent adaptive role in the regulation of neural communication.

### Diffusion measurements in the brain extracellular space

Radiotracers were a preferred early tool for evaluating bulk extracellular diffusion in the brain. When radioactive markers are applied through a lateral ventricle, the decay of radioactivity with increasing distance from brain tissue surface gives an estimate of the apparent diffusion coefficient [50]. This approach has provided important measures of drug dispersion in the nervous tissue, but it can only evaluate bulk diffusion in selected brain regions. A breakthrough came with the introduction of the point-source iontophoresis technique, which used a pair of microelectrodes placed ∼100 μm apart in conjunction with the cell-impermeable tetramethylammonium ion (TMA^+^) as a diffusing probe [27,51]. This technique has been used extensively in different brain areas, both *in vitro* and *in vivo* [4] giving values of α in the range 0.15–0.20 and λ ∼1.6 throughout many brain areas, with some regional variations [52–54].

Subsequent integrative optical-imaging techniques assessed diffusion by analysing the spatial profiles of fluorescent molecules or particles ejected from a pipette tip [55]. Not only did this approach greatly extend the range of available probes, it also enabled real-time registration of diffusion anisotropy in brain neuropil [54,56]. The use of quantum dots (QD; Box 1) as an extracellular fluorescent marker has indicated that extracellular gaps in the brain could be as wide as 30–60 nm [57], a surprisingly large distance compared to the 15–25 nm routinely deduced from electron micrographs [35,43,47].

Another image-deconvolution approach has utilised laser-scanning microscopy to monitor fluorescence recovery after photobleaching (FRAP; Box 1). The rate at which an extracellular fluorescent indicator fills a small photobleached tissue volume provides a measure of extracellular diffusion rates *in vivo* [58,59]. An important improvement came with two-photon excitation microscopy. Because such excitation occurs only within a thin (∼1 μm) focal plane, recorded fluorescence provides a reliable readout of the extracellular dye concentration, thereby removing the need for analytical deconvolution [60]. The combination of FRAP, point injection, and two-photon excitation has yielded λ = 2.14 for the diffusion value of large proteins in the brain [61]. A similar imaging methodology has been used to evaluate the diffusion of small fluorescent molecules on a scale of ∼10 μm [62], giving λ = 1.59 for synaptic neuropil in the hippocampus [18]. Most recently, the development of microfibre-optic spot-imaging has improved resolution to just a few microns, evaluating α in several brain regions (values 0.16–0.22) based on concentration-dependent self-quenching [63] and volume partitioning [64] of extracellular indicators.

These advances have significantly increased our understanding of the extracellular mobility of common neurotransmitters. The diffusion coefficients (*D)* for glutamate (based on glutamine measurements) and GABA at 25^o^C in water are 0.76 μm^2^/ms and 0.83 μm^2^/ms, respectively [65]. However, viscosity of cerebrospinal fluid [28] is ∼10% higher than that of water [66]. Further, water viscosity decreases by ∼26% at 35^o^C compared to 25^o^C [67]. Taken together, these data predict that in a cerebrospinal medium, *D* for glutamate and GABA at near-physiological temperatures are 0.86 and 0.94 μm^2^/ms, respectively. Given λ = 1.6, the apparent macroscopic diffusion coefficients for glutamate and GABA in the brain neuropil are therefore reduced λ^2^–fold, giving 0.32 μm^2^/ms and 0.37 μm^2^/ms, respectively.

Notwithstanding these advances, neurotransmitter mobility on the nanoscale, especially inside synaptic clefts, remains poorly understood. The only experimental estimate of the intra-cleft diffusion coefficient for glutamate, 0.33 μm^2^/ms, has been obtained by analysing changes in synaptic AMPAR currents following controlled retardation of extracellular diffusion by dextran [46]. The estimated value suggests a substantial reduction in diffusion, probably due to macromolecular obstacles, inside the cleft. Intriguingly, this estimate is generally consistent with a fourfold retardation of ion mobility measured directly inside clefts that are formed via cell adhesion between cellular membranes and substrates *in vitro* [68]. A promising optical approach that could allow evaluation of quasi-instantaneous molecular mobility *in situ* is time-resolved fluorescence anisotropy imaging (TR-FAIM) [69,70]. This method uses the fact that molecular mobility directly reflects the extent to which the molecular emission plane diverges from the excitation plane over the 1–3 ns lag between excitation and emission events.

## Lateral traffic in synaptic and perisynaptic membranes

### Membrane diffusion of synaptic receptors: a key transport mechanism

In 1974 it was demonstrated that the protein rhodopsin is subject to constant motion in the fluid matrix of the lipid bilayer [71]. It has subsequently been shown that, as with many other proteins, neurotransmitter receptors diffuse in the plane of neuronal plasma membranes by oscillating between confined and free Brownian motion. This diffusion can provide a mechanism for receptor recruitment to, and removal from, synapses [72–75].

The processes that control the synaptic delivery and removal of neurotransmitter receptors have been the focus of intense investigation for over a decade. Significant advances have been achieved, not least the realisation that modulation of lateral membrane diffusion is fundamental to receptor trafficking and retention in the membrane. Because synaptic AMPARs mediate nearly all fast excitatory synaptic transmission, and changes in their number, composition and/or properties mediate synaptic plasticity, these receptors have been studied in particular detail. Diverse mechanisms have been proposed for AMPAR delivery to synapses under basal and stimulated conditions. For example, experiments using photoreactive antagonists and electrophysiological recordings were interpreted to suggest that AMPARs are only exocytosed into the somatic plasma membrane and then diffuse laterally within the dendritic membrane to synapses [76]. A different conclusion has been drawn from real-time imaging approaches; these suggest that AMPARs undergo intracellular transport [77] and are inserted either directly into the PSD [78] or into the dendritic shaft plasma membrane close to, but not in, dendritic spines [73,79,80]. Notwithstanding these apparently disparate results for some aspects of AMPAR trafficking, it is now widely accepted that lateral diffusion in the plasma membrane is a major contributor to the exchange of receptors in and out of the PSD [74,81–84].

The membrane topology of spines has been reported to restrict lateral diffusion [85], but in cultured hippocampal neurones synaptic activity can recruit surface-expressed AMPARs to the spines from the shaft via membrane flow that overrides lateral diffusion barriers to enhance membrane-protein delivery into spines [73]. Another recent report investigated AMPAR lateral movement and exocytosis in hippocampal slices during long-term potentiation (LTP). AMPAR lateral diffusion from the extrasynaptic spine surface enhances the number of receptors at the PSD, whereas AMPARs are exocytosed into dendritic membrane to replenish local extrasynaptic receptor reserves [80]. Overall, the evidence suggests that some, and very probably most, membrane proteins are delivered to the PSD via a process involving exocytosis of membrane proteins in the vicinity of dendritic spines followed by their subsequent lateral diffusion to the PSD.

Inhibitory glycine receptors (GlyRs) and GABA_A_Rs are subject to synaptic, perisynaptic, and extrasynaptic membrane diffusion domains [86,87]. At extrasynaptic sites both GlyRs and GABA_A_Rs diffuse relatively freely but display decreased diffusion and confinement at inhibitory synapses and at gephyrin clusters. The slower diffusion at synapses is determined by the presence of obstacles as well as specific binding to scaffolding proteins or cytoskeletal components [86–88]. This leads to subtle differences in the effects on diffusion. Specific interactions with proteins such as gephyrin lead to receptor retention and increased density within defined areas [86,87]. In turn, gephyrin clustering involved in GlyR trafficking can be regulated by integrin-mediated interactions with the extracellular matrix [89]. Obstacles, on the other hand, hinder diffusion and nonspecifically alter the dynamics of receptor lateral diffusion [75]. These changes are activity-dependent: for example, GABA_A_R diffusion dynamics are regulated by neuronal activity in a Ca^2+^- and calcineurin-dependent manner. Normally stable synaptic GABA_A_R clusters undergo rapid and reversible dispersal [90,91].

### Lateral diffusion in the plasma membrane: basic constraints

**Figure 2:**
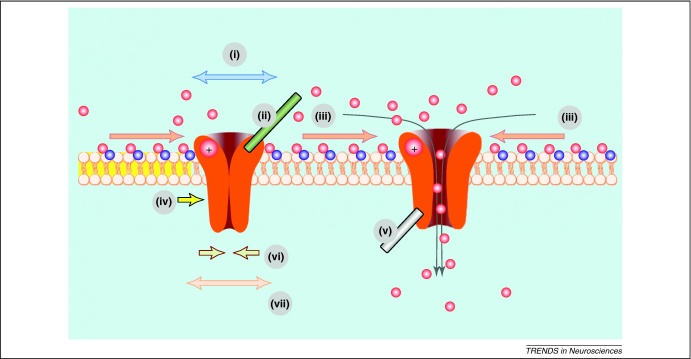
The principal forces affecting receptor mobility in the membrane. Receptor-channels (closed on the left, open on the right) are exposed to forces that can include: **(i)** Brownian motion; **(ii)** Interactions with ECM molecules and other molecular interactions in the extracellular space; **(iii)** Sub-membrane electro-osmotic drag arising in the presence of a receptor current (illustrated by inward current through the open receptor on the right, red and blue balls depict positive and negative ions/charges, respectively); **(iv)** Forces at the interface of membrane areas that have different fluidities/viscosities (such as lipid raft borders, depicted by yellow) and/or membrane flow; **(v)** Interactions with cytoskeletal elements and other intracellular interactions (e.g. immobilisation of AMPARs upon Ca^2+^ influx, probably due to actin stabilisation [107]); **(vi)** Viscous in-membrane friction which prevents free movement; **(vii)** Electric interactions (repulsion or attraction) due to uncompensated electrophoretic charges and ion currents.

Several factors determine the motility of proteins within the neuronal plasma membrane. A major influence is the viscosity of lipid membranes (in the range 0.1–0.3 Pa s; 100–300-fold the viscosity of water). Membrane viscosity depends on the underlying fatty acid content [92] and has long been known to vary substantially in different regions of the cell [93,94]. Single-particle tracking has recently revealed that postsynaptic membranes exhibit lipid-raft-like properties in which the density of local diffusion obstacles depends on filamentous actin [95]. Extramembrane influences, such as those exerted by the extracellular matrix (ECM), the cytoskeleton or by intracellular protein scaffolding could contribute substantially to membrane mobility of proteins [1,96] (Figure 2). Indeed, tracking the GluN1 subunit of the NMDAR using antibody-coupled QDs recently revealed that matrix metalloproteinase 9 (an endopeptidase involved in the degradation of ECM proteins) affects receptor diffusion in cultured hippocampal neurons [97].

**Figure 3:**
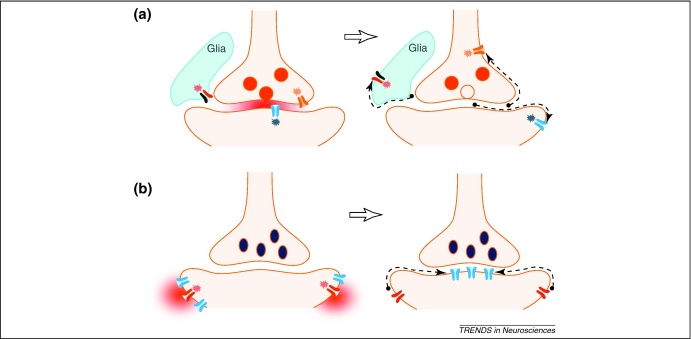
Interplay between diffuse neurotransmitter signals and membrane receptor trafficking can deliver molecular messages to a targeted cellular domain. **(a)** Receptor activation by local release of neurotransmitter (left) can increase the mobility and range of lateral diffusion for the activated receptor (right), as reported for mGluR5 [98]. This mechanism could in principle deliver receptor signals to cellular sites unreachable by neurotransmitter. **(b)** Conversely, sparsely distributed (extra-synaptic) GABA_A_Rs (left; depicted in blue) can cluster at inhibitory synapses (right) in response to activation of nearby NMDARs [91] (left; glutamate cloud and NMDARs depicted in red), thus delivering the receptor signalling machinery to the target site.

Importantly, receptor mobility can be affected by conformation changes induced by ligand binding. Examples include activation to increase the rate of mGlu5 receptor diffusion [98], increased synaptic anchoring of AMPARs following presynaptic glutamate release [99], and increases in the mobility of postsynaptic GABA_A_Rs in parallel with neuronal activity [90]. A recent study has suggested that NMDAR activation is mechanistically involved in inducing GABA_A_R clustering [91]. That the receptor distribution in plasma membranes could be regulated by transmitter actions suggests a potentially important mechanism of signal transfer in which activated receptors can deliver their downstream message to sites unreachable by neurotransmitters. Such a mechanism could thus help to extend the spatial domains of extracellular and intramembrane diffusion-dependent signalling (Figure 3).

These phenomena could also be synapse-specific: for example, the diffusion of α7-subunit-containing nicotinic acetycholine (nACh) receptors in cultured hippocampal neurons was found to be significantly slower at glutamatergic synapses than at GABAergic perisynaptic sites [100]. Furthermore, probing the recovery of NMDAR responses from irreversible pharmacological blockade has suggested higher mobility of presynaptic compared to postsynaptic receptors [101]. The molecular machinery controlling the use-dependent mobility of synaptic receptors remains a subject of intense investigation [3,6].

Membrane surface geometry also influences lateral receptor trafficking. For example, the diffusion of AMPARs is restricted at the neck of dendritic spines [85]. Computer simulations indicate that the diffusion behaviour of molecules in membranes could differ substantially from the diffusion in the lumen under similar conditions [102]. Intriguingly, membrane curvature has been suggested to have highly non-linear effects on the boundaries of cell-membrane domains that differ in fluidity [103]; this is likely to impact upon the dwell-time of locally trapped protein molecules. In addition, optical microscopy routinely deals with planar projections of 3D structures, and therefore both particle-tracking and photobleaching data could require stereological correction to avoid bias in measuring distances and speeds.

Much less is known about the relationship between lateral receptor movements and local electric fields. Substantial evidence indicates that many membrane proteins accumulate cathodally when cells are exposed to a physiologically relevant electric field [1,104]. This suggests that a positive electrophoretic charge on an extracellular domain is not uncommon for membrane receptors. If this is the case for synaptic receptors, their perisynaptic mobility could be influenced not only by intra-cleft electric fields of synaptic currents [49] but also by electro-osmotic drag (which is prominent in the sub-membrane layer) [105] and electrostatic repulsion [106] (Figure 2). The adaptive significance of these electrodiffusion phenomena in the functioning of central synapses remains to be determined.

### Membrane movement of synaptic receptors: a role for rapid use-dependent control of transmission

**Figure 4:**
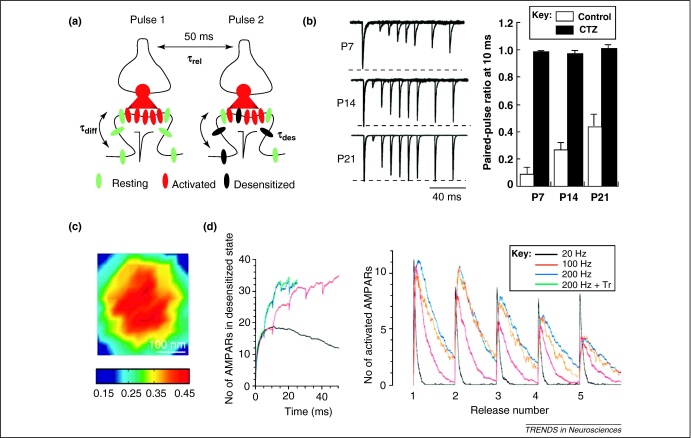
Lateral diffusion can counter desensitisation of synaptic AMPARs during repetitive releases. **(a)** Schematic showing that following glutamate release (red cloud) the activated AMPARs (desensitised over time τ_des_) are exchanged (over time τ_dif_) for resting (naive, non-activated) receptors (before the next release event in time τ_rel_); (adapted, with permission, from [107]). **(b)** In the calyx of Held, AMPAR recovery from desensitisation shortens drastically from P7 to P21, as shown by paired-pulse experiments (left), in which AMPAR desensitisation can be suppressed pharmacologically by cyclothiazide (CTZ, right); (reproduced, with permission, from [110]). **(c)** Simulated profile of desensitised AMPARs inside the synaptic cleft 1 ms post-release predicts 40–50% receptor desensitisation; colour scale shows receptor fraction (reproduced, with permission, from [13]). **(d)** Time-course of AMPAR desensitisation (left) and activation (right) as predicted by a Monte Carlo model [18] during repetitive releases of glutamate at 20, 100, and 200 Hz suggests a significant impact upon synaptic responses (with little effect from transporters, Tr); (reproduced, with permission, from [18]).

Lateral mobility of synaptic receptors provides a basic mechanism by which their local numbers and therefore the synaptic signal can be modified. Intriguingly, lateral exchange between activated (and thus desensitised) and non-activated postsynaptic AMPARs has been proposed to counter desensitisation-dependent depression of synaptic responses during repetitive release [107] (Figure 4a). This hypothesis is conceptually important because it directly associates AMPAR mobility with a fundamental property of excitatory synapses. Several conditions need to be met for this mechanism to have a substantial functional impact. First, the characteristic lifetime of AMPAR desensitisation (τ_des_) should exceed that of receptor dispersion as a result of diffusion (τ_diff_) which, in turn, should be greater than the interval between releases (τ_rel_) (i.e. τ_des_ > τ_diff_ > τ_rel_; Figure 4a). Second, glutamate release should desensitise a significant proportion of local AMPARs: otherwise, diffusion exchange will have little impact upon the availability of non-desensitised receptors to the released neurotransmitter.

The value of τ_des_ depends on the glutamate concentration waveform and on the receptor desensitisation properties *per se*. Desensitisation depends on the receptor subunit composition; this can vary substantially between different types of synapse and also during development [108]. For instance, τ_des_ in hippocampal CA3 pyramidal cells is threefold higher than in dentate gyrus basket cells (∼15 and ∼6 ms, respectively) [109], whereas in the cerebellum or in the auditory system AMPARs recover from desensitisation exceptionally rapidly (1–3 ms), thereby allowing for high bandwidth transmission [14,15]. Remarkably, during maturation of the calyx of Held, synaptic AMPARs show desensitisation lifetimes up to 1 s at postnatal day 7 (P7), 26 ms at P14, and 16 ms at P21 (Figure 4b) [110]. These profound changes have been related to a decrease in the GluA1/GluA4 content ratio of AMPARs during maturation [110]. The latter is consistent with the slow desensitisation recovery (∼111 ms) of receptors composed only of GluA1 [111].

Although there is no direct experimental evidence profiling AMPAR activation and desensitisation inside the cleft, detailed simulations predict that AMPARs are activated principally within a hotspot located opposite the presynaptic release site [11–13,112]. Consistent with electrophysiological observations, these models estimate that AMPAR activation peaks at 60–75% and desensitisation reaches ∼40–50% of the local synaptic AMPAR population (Figure 4c). When glutamate is released repeatedly from the same spot, classical AMPAR kinetic values [113] predict 15–20% paired-pulse depression at 50 ms inter-pulse intervals [18] (Figure 4d). However, this depression can be substantially larger if local AMPARs contain a high proportion of GluA1 subunits [110,111].

Recent data on single-particle tracking of AMPARs indicate a diffusion coefficient of 80 nm^2^/ms in perisynaptic membranes and 15 nm^2^/ms inside the synapse [95], although earlier findings estimated this value in neuronal dendrites to be in the range of 100–250 nm^2^/ms [107]. These data suggest that a characteristic time for AMPARs to disperse from the 100–150 nm-wide hotspot (radius *R* = 50–75 nm) is *R*^*2*^*/4D* = 40–80 ms for the lowest estimated diffusion coefficient value. Therefore, with an immobile release site and a specific AMPAR subunit composition, lateral AMPAR diffusion could successfully counter receptor desensitisation during repetitive glutamate release [107]. An important question remains regarding how often these conditions occur *in vivo* to enable this mechanism to have a substantial physiological impact.

## Concluding remarks

Extracellular diffusion of neurotransmitters and the lateral diffusion of receptors in the synaptic environment form the basis for rapid signalling in brain circuits. In recent years our perception of the synapse has changed from being considered as a relatively stable connection undergoing use-dependent changes on the scale of seconds or minutes to a highly mobile structure shaped in large part by the millisecond-scale dynamics of its molecular components. This change of view has implications for the basic functional features of neural connections.

First, vigorous constitutive trafficking of synaptic components suggests that a synaptic ‘resting state’ is only a relative notion, and that modulation of synaptic efficacy is likely to reflect a change in the rate of the underlying dynamics rather than a switch from an inactive mode to an active one. Not only does the constitutive activity enable a more rapid response of the synaptic system to an external influence, but it also provides a highly flexible dynamic range of such responses. Second, because dynamic components of neural connections are subject to multiple intra- and extracellular influences, the outcome of synaptic activity can be finely tuned by the local environment and network activity in real time. Finally, this highly mobile and inclusive system provides several different potential mechanisms by which a memory trace could be generated and stored, be it via changed amplitude of individual synaptic responses [such as during synaptic LTP or LTD (long-term depression)] or through more complex effects such as modifications in response variability, kinetics, or burst-induced plasticity. These multiple interactions need to be considered in their evolving dynamics to understand what shapes and modifies elementary signals in brain circuits, and in both health and disease (Box 2).

**Glossary**

**Hebbian learning:** the principle originally introduced by Hebb [124]. This describes a basic mechanism for associative synaptic plasticity in which a long-lasting increase in synaptic transmission strength arises either from the persistent activation of a particular connection or from coincidence of postsynaptic and presynaptic excitatory events.
**Intra-cleft electric fields:** these arise when electric current flows from outside the synaptic cleft through open ion channels in the synaptic membranes. This field reflects the voltage drop between the edge and the centre of the cleft and could affect charged neurotransmitter molecules or electrophoretic charges on extracellular domains of receptor proteins.
**Electro-osmotic drag:** the viscous force arising near cell membrane surfaces in the presence of a local ion current. Because the surface of most live cells is negatively charged this attracts a quasi-equilibrated layer of mobile cations. When a local ion channel is open, thus generating electric current, this cation layer can move along the surface and drag the adjacent molecular structures, including mobile membrane proteins, alongside.
**Photobleaching:** the irreversible loss of fluorescence following excitation. Its rate or probability depends on the wavelength, intensity and mode of excitation. Although normally considered as a drawback of fluorescent probes, local photobleaching can be used to measure the mobility of non-photobleached molecules diffusing into an area containing bleached molecules.
**Quantum dots (QDs):** these are roughly protein-sized (5–50 nm) semiconductor nanocrystal fluorophore particles. Their fluorescence depends on their crystal properties and they are bright and highly resistant to photobleaching.
**Receptor desensitisation:** a temporary loss of sensitivity or responsiveness to a ligand following receptor activation. The degree of desensitisation and recovery from it can depend on local conditions. In general, desensitisation helps to prevent excessive activation of receptors.
**Signal waveform:** generically describes the evolvement of a functional variable in time and space, such as the rise and fall of ion current or molecular concentration in 3D. Signal shape normally refers to the time domain only (e.g. the kinetics or the time-course of an event).
**Stereological correction:** addresses a common measurement bias in microscopy when most observations represent planar (2D) projections of 3D structures. Several different theoretical approaches and empirical procedures have been used to account for such bias.
**Time-resolved fluorescence anisotropy imaging (TR-FAIM):** this relies on the fact that a stationary fluorophore excited by polarised light will emit polarised light (normally in the same polarisation plane). If the fluorophore is moving the emitted light will have a different orientation from the excitation light. Therefore, fluorescence intensities measured in polarisation planes parallel and perpendicular to that of the excitation light can be used to gauge the rotational mobility of the fluorophore.
**Tortuosity (λ):** represents the hindrance imposed on the diffusing molecules by the tissue, cellular structures or large macromolecular obstacles in comparison with an obstacle-free medium.
**Two-photon excitation microscopy:** this uses the principle that two lower-energy (red-shifted) photons can be absorbed simultaneously by a fluorophore to initiate emission of one higher-energy photon. Under a microscope, this restricts excitation to a thin focal plane and, used in conjunction with infrared light to minimise light scattering, allows fluorophores to be monitored up to a depth of ∼1 mm.
**Extracellular volume fraction (α):** the proportion of tissue volume occupied by the extracellular space.

## Box 1 Monitoring the lateral trafficking of proteins in neuronal membranes

Several experimental approaches have been used to monitor the diffusion of specific proteins within and between membrane compartments, each of which has advantages and caveats.

**Single particle tracking (SPT):** allows near real-time imaging of a probe coupled to the extracellular domain of the transmembrane protein (usually a receptor) of interest via an antibody, which recognises either a recombinant epitope-tagged or native protein. Although it can be technically challenging, SPT allows the diffusion of individual proteins to be tracked without population averaging. Initial probes were latex beads, which can be imaged by phase-contrast microscopy but which are comparatively bulky (>100 nm) compared to the 20–40 nm wide synaptic cleft [86,98]. Antibodies directly coupled to fluorescent organic dyes (e.g. Cy5; ∼30 nm) have also been used, but these emit relatively little fluorescence and photobleach rapidly [81]. The most powerful tools currently available for SPT are quantum dots (QDs) [114], and their use has provided new insights in the regulation of lateral diffusion. For example, QDs allow direct visualisation of the movement of proteins into defined membrane domains such as the PSD [81,115] and have provided the first clear demonstration that receptors can enter and exit the postsynaptic site by lateral diffusion [87]. Furthermore, this approach has enabled two important discoveries: (i) that AMPAR anchoring at synaptic sites depends both on presynaptic activity [99] and the interaction with PSD-95 via Stargazin [115], and (ii) that NMDAR diffusion and synaptic recruitment is regulated by the ECM protein Reelin [115]. Similarly, QD approaches have been used to demonstrate that GABA_A_Rs and glycine receptors also undergo activity-dependent lateral diffusion to and from the synapse [72,87,90,91].

**Fluorescence recovery after photobleaching (FRAP):** this technique utilises the fact that under intense or prolonged light exposure some fluorophores undergo photobleaching. FRAP was first used in the 1970s to monitor diffusion in cell membranes [116] and has been used extensively ever since. Selected membrane areas are photobleached and the kinetics of fluorescence recovery (e.g. half-time) indicates the mobility of the fluorescent target protein. The proportion of local fluorescence that fails to recover signifies the fraction of protein that is immobile. The most frequently used fluorophores for FRAP are variants of green fluorescent protein (GFP) cloned into the coding sequence of proteins of interest. In particular, the pH-sensitive GFP variant superecliptic pHluorin (SEP) [82,117] has been used to report only surface-expressed receptors [73,82]. For reliable data acquisition it is necessary to obtain a fluorescence signal exclusively from a single layer of plasma membrane; this is complicated because dendrites are cylindrical structures and spines are constantly mobile and have various shapes. In addition, morphology can complicate the determination of the area of membrane bleached, and fluorescence recovery itself can be affected by membrane shape [118]. Notwithstanding these caveats, FRAP does not require highly specialised microscopy equipment and is therefore a relatively accessible method for determining the diffusion of populations of proteins in the plasma membrane.

**Fluorescence correlation spectroscopy (FCS):** the analysis of the fluctuation of fluorescence intensity, where fluctuations represent the variation in the mean number of fluorescent proteins [119,120]. When tagged proteins or lipids pass through the light-excited membrane domain they produce fluctuations in the fluorescence within that domain; the more proteins diffuse the greater the signal fluctuates. This allows accurate determination of cell membrane structures, for example spines, at very high resolution [121–123]. To date, however, few studies have used FCS to monitor the diffusion properties of specific membrane proteins.

## Box 2 Outstanding questions

### Mobility of signalling molecules in the extracellular space

• Signalling molecules move rapidly between adjacent cell membranes, but do they move as fast as in a free medium? How long do they dwell inside the synaptic cleft?
• The ECM provides structural support for synaptic elements and mediates important signalling exchanges between synapses and astroglia. What is its role in shaping synaptic environment and in controlling diffusion-dependent neurotransmitter signalling?
• The synaptic cleft is packed with macromolecules. How dynamic is cleft architecture and how does it affect the access of neurotransmitters to synaptic and extrasynaptic receptors?
• What are the roles and major determinants of gaseous signalling molecules, such as nitric oxide (NO), in the synaptic environment?
• Extracellular diffusion properties change dramatically during ischaemia, epileptic seizures and other pathologies. Are such changes involved in the underlying disease mechanisms? Can they be used to detect and/or counteract a developing dysfunction?

### Lateral mobility of proteins in neuronal membranes

• What is the relationship between intracellular transport aided by motor proteins and ‘free’ membrane diffusion – can the cell control this relationship depending on physiological circumstances?
• How do dynamic changes in neuronal membrane shape, such as local curvature, influence the lateral diffusion of receptors?
• Neuronal membranes could show distinct basic properties, such as composition and fluidity, in different conditions. Do neurons control such properties in different regions of the cell and thus regulate membrane traffic? Could lipid rafts represent a ‘picket fence’ when and where required?
• ECM proteins interact dynamically with membrane proteins. Can this interaction be controlled by neuronal activity?

### Dynamic organisation of the synaptic environment: a role for synaptic function

• Most detailed knowledge about membrane organisation and protein trafficking has been obtained from *in vitro* experiments. How much does this knowledge apply to the working of synaptic machinery *in vivo*?
• Molecular dynamics in the synaptic environment are likely to change during development and ageing. What are the molecular mechanisms underlying such changes?
• Changes in the molecular mobility of signalling molecules or synaptic components could be either a cause or a consequence of pathological synaptic changes in different diseases. What are the molecular targets in the underlying mechanisms that are potentially suitable for therapeutic intervention?
